# Increased blood–brain barrier permeability to water in the aging brain detected using noninvasive multi‐TE ASL MRI

**DOI:** 10.1002/mrm.28496

**Published:** 2020-09-10

**Authors:** Yolanda Ohene, Ian F. Harrison, Phoebe G. Evans, David L. Thomas, Mark F. Lythgoe, Jack A. Wells

**Affiliations:** ^1^ UCL Centre for Advanced Biomedical Imaging, Division of Medicine University College London London United Kingdom; ^2^ Neuroradiological Academic Unit, Department of Brain Repair and Rehabilitation UCL Queen Square Institute of Neurology University College London London United Kingdom; ^3^ Dementia Research Centre UCL Queen Square Institute of Neurology University College London London United Kingdom; ^4^ Wellcome Centre for Human Neuroimaging UCL Queen Square Institute of Neurology University College London London United Kingdom

**Keywords:** aging, aquaporin‐4, blood–brain barrier, blood–brain interface, arterial spin labeling, water permeability

## Abstract

**Purpose:**

A fundamental goal in the drive to understand and find better treatments for dementia is the identification of the factors that render the aging brain vulnerable to neurodegenerative disease. Recent evidence indicates the integrity of the blood–brain barrier (BBB) to be an important component of functional failure underlying age‐related cognitive decline. Practical and sensitive measurement is necessary, therefore, to support diagnostic and therapeutic strategies targeted at maintaining BBB integrity in aging patients. Here, we investigated changes in BBB permeability to endogenous blood water in the aging brain.

**Methods:**

A multiple‐echo‐time arterial spin‐labeling MRI technique, implemented on a 9.4T Bruker imaging system, was applied to 7‐ and 27‐month‐old mice to measure changes in water permeability across the BBB with aging.

**Results:**

We observed that BBB water permeability was 32% faster in aged mice. This occurred along with a 2.1‐fold increase in mRNA expression of aquaporin‐4 water channels and a 7.1‐fold decrease in mRNA expression of α‐syntrophin protein, which anchors aquaporin‐4 to the BBB.

**Conclusion:**

Age‐related changes to water permeability across the BBB can be captured using noninvasive noncontrast MRI techniques.

## Abstract 2

Click here for author‐reader discussions

## 1 INTRODUCTION

The blood–brain barrier (BBB) is a dynamic and regulatory interface that protects the brain parenchyma from deleterious infiltration.^1^ Its composition includes endothelial cells, tight junction proteins, pericytes, and aquaporin‐4 (AQP4) water channels that reside on astrocytic end feet. Dysfunction of the BBB is an early occurring feature of age‐related neurodegenerative pathologies, such as Alzheimer disease (AD).^1^, ^2^, ^3^ Recent data have identified changes in BBB integrity to be associated with early age‐related cognitive dysfunction in the human brain, relative to more established biomarkers such as tau and amyloid.^4^ This finding suggests that dysfunction of the BBB, such as changes to BBB permeability, is a high‐priority target for practical and sensitive measurement of neurodegenerative processes.

The structural integrity of the BBB is typically assessed by capturing the egress of exogenous contrast agents from the blood into the brain parenchyma. In patients, this usually involves intravenous delivery of a gadolinium‐based contrast agent (GBCA) with dynamic T_1_‐weighted MRI to assess BBB‐mediated extravascular GBCA accumulation (known as dynamic contrast‐enhanced MRI [DCE‐MRI]^5^). These techniques have shown promising results in detecting an increased BBB permeability in patients with the early stages of cognitive impairment in AD.^6^ Further studies using GBCA in a novel MRI technique sensitized to vascular water exchange have also reported increased water permeability across the BBB in a rat model of AD, when the DCE‐MRI permeability measurement did not yet show significant differences between the animal groups.^7^ This would imply that measures of water permeability enable measurements of subtle changes to the BBB that occur in the early stages of neurodegenerative disease.

Brain water homeostasis is maintained by tight regulation of water transport mechanisms across the BBB; its dysfunction manifests pathologically in several brain pathologies, such as cerebral edema,^8^ meningitis,^9^ and brain tumors.^10^ MR techniques targeting the transfer of endogenous water molecules across the BBB have demonstrated sensitivity to patients with the presence of vascular risk factors,^11^ sleep apnea,^12^ brain tumors,^10^ and postischemic brain tissue in a rat model of stroke.^13^ Broadly, these noninvasive methods use modified arterial spin‐labeling (ASL) MRI sequences, that eliminate the need for a GBCA. The adapted ASL sequences aim to separate the intravascular (IV) and extravascular (EV) origin of a labeled bolus of blood water as it is delivered to the brain tissue.^14^, ^15^, ^16^, ^17^, ^18^ Therefore, water permeability measurements could be useful for better understanding the subtle changes that occur in AD, using the small endogenous water molecules to probe the integrity of the BBB.

Despite age being the primary risk factor for dementia, there have been no prior studies principally designed to investigate changes in BBB water permeability with age. Our previous work demonstrates the sensitivity of a multiple‐echo‐time (multi‐TE) ASL technique to the presence of AQP4 water channels, an important route for water transport across the BBB.^19^, ^20^ It is known that AQP4 water channels are upregulated in the aging brain, which may be an adaptive response to maintain water homeostasis.^21^ Here we describe the application of multi‐TE ASL methods to assess BBB permeability to water in a mouse model of aging. We aim to establish whether BBB permeability to water changes as the brain ages, together with the upregulation of AQP4, and if so, whether these differences can be detected by noninvasive, clinically relevant MRI techniques.

## 2 METHODS

### 2.1 Experimental protocol

All experiments were performed in accordance with the European Commission Directive 86/609/EEC (European Convention for the Protection of Vertebrate Animals Used for Experimental and Other Scientific Purposes) and the United Kingdom Home Office Animals (Scientific Procedures) Act (1986). All mice were acclimatized in an animal house, prior to data acquisition, with a 12‐hour light/12‐hour dark cycle with food and water provided *ad libitum*.

Eight male C57Bl/6JRj mice at 27 ± 1 months old (29.6‐37.5 g; henceforth referred to as “aged”) and 10 male C57Bl/6JRj mice at 7 ± 1 months old (30.1‐35.7 g; henceforth referred to as “adult”) were used in this study. All mice were induced with 2% isoflurane anesthetic in a mixture with room air at 1.0 L/min, which was manually adjusted between 1.75%‐1.5% to maintain the respiration rate at approximately 100 bpm, which was measured using a pressure pad and monitored throughout the scan. A rectal probe (SA Instruments, Stony Brook, NY) was used to monitor the core body temperature, which was maintained at 37.0 ± 0.5°C via an adjustable water bath supplied to a mouse heating pad (Bruker BioSpec; Bruker, Kontich, Belgium).

Images were acquired on a horizontal‐bore 9.4T Bruker preclinical system (BioSpec 94/20 USR; Bruker) using a 440‐mT/m gradient set with an outer and inner diameter of 205 mm and 116 mm, respectively (BioSpec B‐GA 12S2), a 86‐mm volume transit RF coil, and a four‐channel receiver‐array coil designed for the mouse brain (Bruker). The ASL image acquisition was based on a flow‐alternating inversion recovery sequence with a single‐shot spin‐echo echo planar imaging readout. A single‐slice flow‐alternating inversion recovery protocol was implemented (slice thickness = 2 mm), with a slice selective inversion pulse thickness of 8 mm and a global nonselective pulse (no slice‐select gradient). Imaging parameters were inflow time (TI) = 800 ms and 1500 ms; echo times (TEs) = 8 ms, 10 ms, 12 ms, 15 ms, 18 ms, 23 ms, 30 ms, 40 ms, 50 ms, 65 ms; pulse repetition time (TR) = 5000 ms; data matrix = 64 × 64; field of view = 25 × 25 mm; slice thickness = 2 mm; partial Fourier = 32 + 4 lines of k‐space; and repetitions = 10. The ASL data at TI = 800 ms and TE = 10 ms were used to estimate cortical cerebral blood flow (CBF). Measurements of the arterial transit time (*δ_a_*) were also captured in the same group of adult and aged mice with a separate acquisition using imaging parameters: TI = 200 ms, 300 ms, 400 ms, 500 ms; TE = 10 ms; TR = 10 000 ms; data matrix = 64 × 64; slice thickness = 2 mm; partial Fourier = 32 + 4 lines of k‐space; and repetitions = 10.

### 2.2 Arterial transit time and cerebral blood flow

The arterial transit time (*δ_a_*) reflects the arrival time of the labeled bolus of blood water to the imaging region of interest and in this work *δ_a_* is estimated using a separate multiple short inflow time (multi‐TI) ASL acquisition. At short TIs the ASL signal, Δ*M*, has, approximately, a linear dependence TI, according to the pulsed arterial spin‐labeling biophysical model^22^:

(1) $$\Delta M=0\;\text{when}\;\;\delta_{a}\;>\;\text{TI}$$

(2) $$\Delta M\left(\mathrm{TI}\right)=A\;\cdot \;\left(TI‐\delta_{a}\right)\cdot \;\exp\left(‐\frac{\mathrm{TI}}{T1_{a}}\right)\;\text{when}\;\delta_{a}<\;\text{TI}$$

where *A* is a constant factor, dependant on CBF (*A* = 2α· *M*_OA_· CBF/λ), T1_a_ is the longitudinal relaxation of the arterial blood (assumed to be 2.4 seconds at 9.4T^23^) and *M*_0A_ is the equilibrium magnetization of the blood.

CBF was assessed by taking the ASL signal, ∆*M* at a short TE^22^:

(3) $$\Delta M=2\;\cdot \;M_{0}\;\cdot \;\left(TI‐\delta_{a}\right)\;\cdot \;\alpha \;\cdot \;CBF\;\cdot \;\left[\frac{\max(0,TI‐\delta_{a})}{TI‐\delta_{a}}\right]\left(\exp\left(‐TI\;\cdot \;R1_{a}\right)\;\cdot \;q_{1}\;\cdot \;\frac{\min\left(0,TI‐\delta_{a}‐\tau \right)}{TI‐\delta_{a}‐\tau }+2\;\cdot \;M_{0}\tau \alpha \;\cdot \;\exp\left(‐TI\;\cdot \;R1_{a}\right)\;\cdot \;q_{2}\frac{\max\left(0,TI‐\delta_{a}‐\tau \right)}{TI‐\delta_{a}‐\tau }\right)$$

(4) $$q_{1}=\exp\left(k\;\cdot \;TI\right)\;\cdot \;\exp\left(‐k\;\cdot \;\delta_{a}\right)‐\frac{\exp\left(‐k\;\cdot \;TI\right)}{k\;\cdot \;\left(TI‐\delta_{a}\right)}$$

(5) $$q_{2}=\exp\left(k\;\cdot \;TI\right)\;\cdot \;\exp\left(‐k\;\cdot \;\delta_{a}\right)‐\frac{\exp\left(‐k\;\cdot \;(\tau +\delta_{a}\right)}{k\;\cdot \;\tau }$$

(6) $$k=\frac{1}{T1_{b}}‐\frac{1}{T1^{\prime }}$$

(7) $$\frac{1}{T1^{\prime }}=\frac{1}{T1}+\frac{\mathrm{CBF}}{\lambda }$$

where *M*_0_ is the equilibrium magnetization from the short TE control signal using the standard recovery model,^24^ TI is the inflow time, $\delta_{a}$ is the arterial transit time, α is the inversion efficiency (0.9), *R*1*_a_* is the longitudinal relaxation rate of the arterial blood, τ is the temporal length of the labeled bolus (previously measured at 1.7 seconds in the mouse brain), and λ is the partition coefficient (assumed to have a value of 0.9 mL/g^25^).

### 2.3 Two compartment model for an index of blood–brain barrier permeability to water

The multi‐TE ASL method is used to separate IV‐EV compartments to model BBB permeability to water. This is achieved by fitting the ASL signal decay to a two‐compartment biexponential model at intermediate TIs (*δ* < TI < *δ* + τ).

(8) $$\Delta M=\Delta M_{\mathrm{IV}}\exp\left(‐\frac{\mathrm{TE}}{T2_{\mathrm{IV}}}\right)+\Delta M_{\mathrm{EV}}\exp\left(‐\frac{\mathrm{TE}}{T2_{\mathrm{EV}}}\right)$$

where Δ*M*_IV_ and Δ*M*_EV_ are intravascular and extravascular ASL signal‐weighting factors, respectively, TE is the echo time, *T*2_IV_ is the T_2_ value of the intravascular compartment, and *T*2_EV_ is the T_2_ value of the extravascular tissue.

The ASL signal weighting factors enable estimation of the intravascular fraction at a given TI:

(9) $$\text{Intravascular fraction}=\frac{\Delta M_{\mathrm{IV}}}{\Delta M_{\mathrm{IV}}+\Delta M_{\mathrm{EV}}}$$

The adapted kinetic perfusion model^26^, ^27^ is applied to ASL signal‐weighting factors to estimate the tissue transit time, *δ*, is described by the following equations:

(10) $$\Delta M_{\mathrm{IV}}=\frac{2M_{0}CBF}{\lambda }\left\{\exp\left(‐TI\times R1_{a}\right)\left[\min\left(\left(\delta_{a}‐TI+\tau ,0\right)‐\delta_{a}\right)‐\left(\min\left(\delta ‐TI+\tau ,0\right)\right)‐\delta \right]\right\}$$

(11) $$\Delta M_{\mathrm{EV}}=\frac{2M_{0}CBF}{\lambda }\left\{\exp\left(‐TI\times R1_{\text{app}}\right)\left[\frac{\exp\left(\min\left(TI,\delta +\tau \right)\Delta R\right)‐\exp\left(\delta \Delta R\right)}{\Delta R}\right]\right\}$$

where *R*1_app_ is the longitudinal relaxation rate of the tissue, here fixed at 1/1.7 seconds,^17^ Δ*R* = *R*1_app_−*R*1*_a_*.

The water exchange time ($T_{\mathrm{ex}}^{w}$) indicates the time for magnetically labeled vascular water to transfer across the BBB into brain tissue after entering the imaging slice:

(12) $$T_{\mathrm{ex}}^{w}=\delta ‐\delta_{a}$$

The exchange time parameter provides a quantitative, surrogate marker of BBB permeability to water.

All measured values and assumed values are displayed in Appendix Tables A1 and A2. A schematic of the temporal length of TI, *δ_a_*, *δ*, and τ can be found in Figure 1 of Wells et al.^17^

**FIGURE 1:**
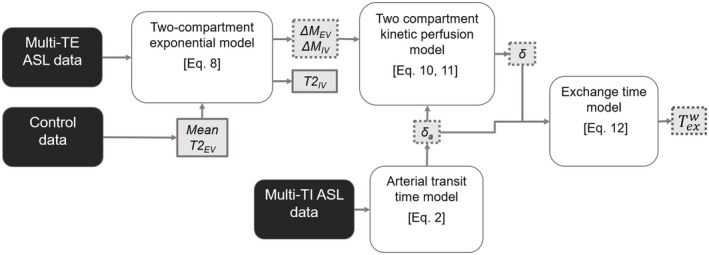
Processing pipeline to measure the exchange time ($T_{\mathrm{ex}}^{w}$) as an index of blood–brain barrier permeability to water using control data to determine extravascular tissue T_2_ (*T*2_EV_), multiple‐echo‐time (multi‐TE) arterial spin‐labeling (ASL) data to determine intravascular/extravascular ASL signal intensities (Δ*M*_IV_/Δ*M*_EV_), intravascular T_2_ (*T*2_IV_) and tissue transit time (*δ*), and multi‐TI (inflow time) ASL data to determine arterial transit time (*δ_a_*). Dotted lines indicate parameters with results for the individual animals and solid lines indicate parameters with result that is shared across the group. Adapted from Ohene et al, 2019^19^

### 2.4 Quantification of mRNA expression of molecular components of the blood‐brain barrier

The noninvasive BBB water permeability measurements were accompanied by assessment of mRNA expression profiles of aquaporin‐4 water channels (*Aqp4*) and α‐syntrophin protein (*SNTA1)* to probe possible age‐related changes to AQP4 polarization. mRNA expression measurements were taken from the cortical brain region of seven C57Bl/6JRj mice at 27 ± 2 months old and seven C57Bl/6JRj at 7 ± 2 months old (the same mice cohort used for MRI BBB water permeability measurements). All mice were euthanized by overdose with an intraperitoneal injection of sodium pentobarbital (10 mL/kg), the brain was removed, hemisected, and the cortex dissected and snap frozen on dry ice.

Total mRNA from each brain region was extracted using the RNeasy Plus Microkit (QIAGEN, Manchester, UK). Total mRNA was converted to cDNA using the QuantiTect Reverse Transcription kit (QIAGEN), which was quantified using the Eppendorf Mastercycler with Realplex software (v1.5; Eppendorf, Stevenage, UK) and the TaqMan Gene Expression Mastermix and Taqman Gene Expression assays (both Applied Biosystems, Warrington, UK). Taqman Gene Expression assays for *Aqp4* and *SNTA1,* and reference housekeeper genes (βACT and GAPDH) were used. Each reaction contained 10‐µL Mastermix, 8‐µL RNase‐free water, 1‐µL gene expression assay, and 1‐µL cDNA, and were performed in triplicate. Each of the mRNA expression levels were determined using the 2^−∆∆Ct^ method^28^ with the normalization factor calculated from the geometric mean of the two housekeeper genes, and internal normalization determined from the adult mice cortical region.

### 2.5 Data processing

All imaging data were processed using MATLAB 2018a (MathWorks, Natick, MA). Automatic scaling of image intensity was applied during the online reconstruction on the Bruker scanner; this was corrected offline to ensure the correct relative signal intensities across different TE and TI values.

Mean ASL images were generated by a pairwise subtraction of the control and labeled images. The processing pipeline as seen in Figure 1 was used to determine cortical BBB permeability to water measurements.^17^ Outliers were determined using ROUT coefficient (Q set at 1%)^29^ and were excluded from further analysis. ∆*M*_IV_ and ∆*M*_EV_ variables (Equations 10 and 11) were constrained to be greater than zero to be physiologically feasible. The separate short multi‐TI data set was used to calculate the arterial transit time (Equation 2). The water exchange time $\left(T_{\mathrm{ex}}^{w}\right)$ was measured by subtracting the arterial transit time from the tissue transit time, to give a surrogate index of BBB permeability to water (Equation 12). Data from one mouse in the adult cohort was excluded from the final analysis because of a technical error that resulted in an incomplete data set.

### 2.6 Statistical analysis

All of the statistical analysis was performed with GraphPad Prism 8 (GraphPad Software, La Jolla, CA). All data are reported as the mean and the associated error (± standard deviation [SD]) across each group. A two‐way ANOVA with Bonferroni’s multiple comparisons test was performed on the intravascular fraction to evaluate the interaction between the two inflow times and the effect of age between the adult and aged mice. A two‐tailed unpaired Student *t* test was performed on the data from the adult and aged mice to compare $T_{\mathrm{ex}}^{w}$, *δ_a_*, CBF, *T*2_EV_, and mRNA expression of *Aqp4* and *SNTA1* measurements. For all tests, *P* < .05 was considered to be a statistically significant result.

## 3 RESULTS

**FIGURE 2:**
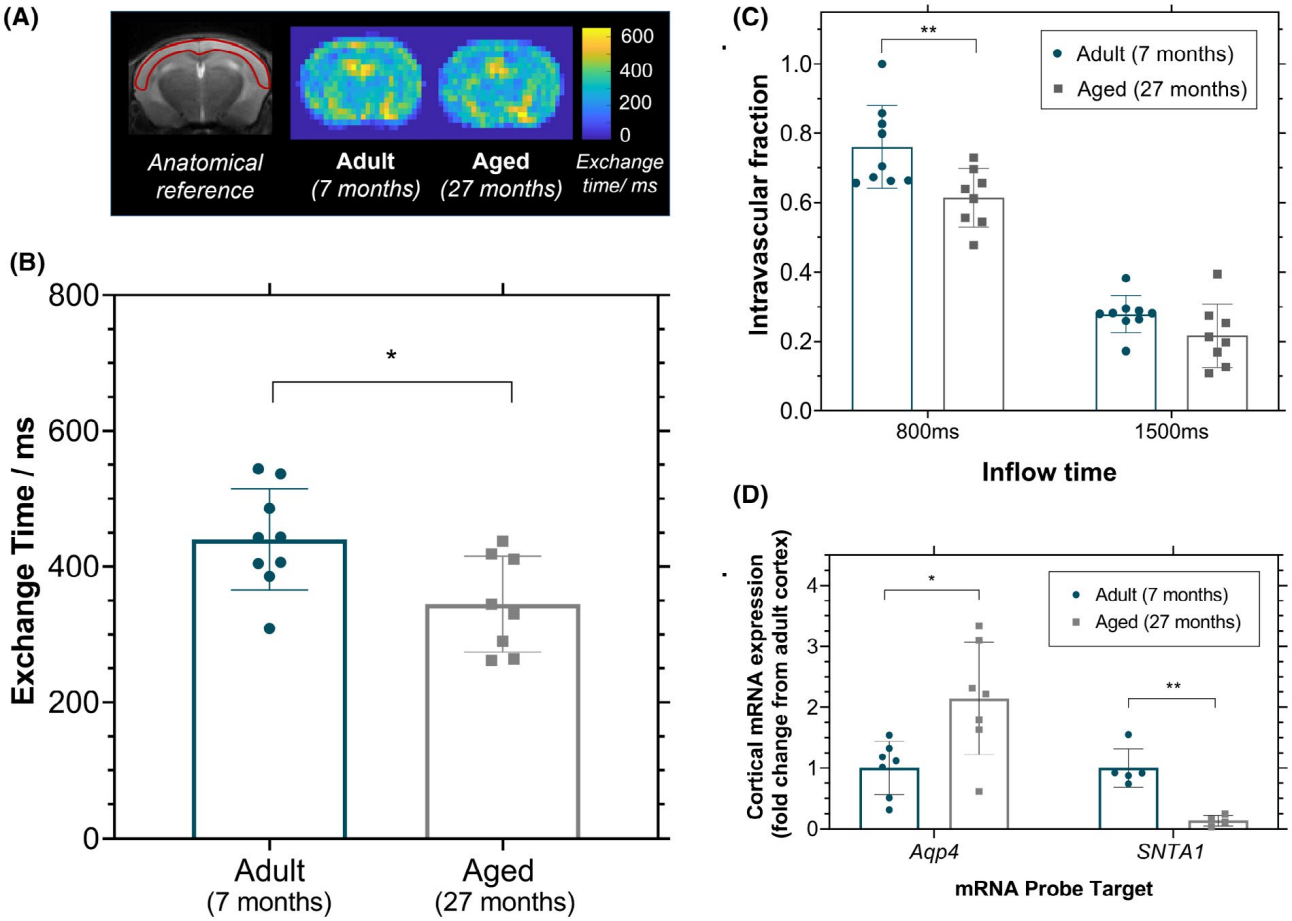
Cortical blood–brain barrier permeability to water. A, Mean exchange time maps for adult and aged mice, with an anatomical reference image on the left with the cortical region of interest in red. B, Cortical exchange time measurements as index for blood–brain barrier water permeability for individual animals. C, The intravascular fraction (Δ*M*_IV_/Δ*M*_IV_ + Δ*M*_EV_) determined for the individual animals at both inflow times. D, Cortical mRNA expression in aging of aquaporin‐4 (*Aqp4*), α‐syntrophin protein (*SNTA1*) reported as fold‐change from the adult cortex. All plots indicate the mean value and associated error (± standard deviation) for adult mice (*n* = 9) and aged mice (*n* = 8). *P < .05; **P < .01

The mean cortical water exchange time was 32% lower in the aged mice (345 ± 71 ms) relative to the adult mice (440 ± 75 ms; *P* = .016; Figure 2A,B). This provides evidence for a faster rate of labeled vascular water flux into the extravascular tissue in the aged mice, indicating an increased BBB permeability to water in the aged brain.

The ASL signal as a function of TE from the cortical brain region was well‐described by a biexponential decay model for each of the mice imaged in this study (Supporting Information Figures S1 and S2). The intravascular fraction was measured to be 0.76 ± 0.12 for adult mice compared with 0.61 ± 0.08 for the aged mice at TI = 800 ms, and 0.28 ± 0.05 for adult mice compared with 0.22 ± 0.09 in the aged mice at TI = 1500 ms (*P* = .006; Figure 2C).

*T*2_IV_ (the T2 of the intravascular component of the ASL signal) in the cortical region was measured to be 13.5 ± 1.2 ms and 11.3 ± 4.1 ms for the aged mice compared with 20.6 ± 1.4 ms and 14.3 ± 4.0 ms for the adult mice, at TI = 800 ms and TI = 1500 ms, respectively (a single *T*2_IV_ value was estimated for each group; therefore, no statistical comparison was performed). The extravascular tissue *T*2_EV_ (taken from the control signal) was measured at 37.1 ± 1.6 ms in the adult mice compared with 35.6 ± 1.5 ms in the aged mice at TI = 800 ms, and 34.5 ± 1.3 ms in the adult mice compared with 32.8 ± 1.6 ms in the aged mice at TI = 1500 ms (*P* = .003).

There were no significant age‐related differences in the arterial transit time in the cortical region in aged mice (125 ± 34 ms) and adult mice (119 ± 45 ms; *P* = .77; Supporting Information Figure S3A). Similarly, there were no significant differences in the CBF measurements in aged mice (188 ± 61 mL/100 g/min), compared with the adult mice (191 ± 71 mL/100 g/min; *P* = .93; Supporting Information Figure S3B).

There was a significant increase in mRNA expression of *Aqp4* in the cortical region of the aged mice (2.1 ± 0.9‐fold change) compared with the adult mice (*P* = .01; Figure 2D). This was accompanied by a marked decrease in *SNTA1* mRNA expression in the aged mice (0.14 ± 0.09‐fold change), which indicates a downregulation of the α‐syntrophin protein in the aging mouse brain, which is in part responsible for anchoring AQP4 to the BBB (*P* = .001, Figure 2D).

## 4 DISCUSSION

Dysfunction of the BBB appears to be a promising candidate as an early predictor of age‐related cognitive decline, independent of the classic pathophysiological hallmarks, amyloid and tau.^4^ As such, there is a need for minimally invasive measurement techniques, sensitive to changes in the structural and functional integrity of the BBB with age. Here we provide evidence for increased water permeability across the BBB in the aged brain detected using a clinically relevant, noncontrast ASL‐based MRI technique.

The mean cortical water exchange time was significantly faster (32%) in the aged mouse brain cortex relative to the adult mice, indicating decreased BBB resistance to water penetration with age. To our knowledge, this is the first demonstration of measurable changes to water permeability across the BBB in the aging brain. An increase in water exchange across the BBB has previously been reported in a rat model of AD, which led the authors to suggest that changes in BBB permeability to water may elucidate early breakdown in the BBB, as the gold‐standard DCE technique was unable to differentiate between the animal groups.^7^ Extending these water permeability studies to include cognitive behavioral tests would provide further insight into whether early changes in BBB water permeability represent an upstream biomarker of disruption to BBB integrity that independently predicts age‐related cognitive dysfunction.

Brain AQP4 proteins are intramembrane bidirectional channels, central to water exchange across the BBB. We have previously demonstrated sensitivity of multi‐TE ASL to the presence of AQP4, recording a 31% longer cortical water exchange time in *Aqp4* knockout mice.^19^ Here, we report the cortical *Aqp4* mRNA expression to be significantly higher (2.1 ± 0.9‐fold increase) in the aged mice. We also observed a marked downregulation (0.14 ± 0.09‐fold change: ~7‐fold decrease) of mRNA expression of *SNTA1* in the aged mouse brain, an anchoring protein of AQP4 to the BBB that gives an index of the extent of AQP4 polarization. We hypothesize that an increase in AQP4 expression and decrease in AQP4 polarization would have opposing effects on the rate of water exchange time, which makes it difficult to predict the overall influence of AQP4 water‐channel proteins on water permeability across the BBB. Therefore, further studies are required to elucidate the precise changes to the structure of the BBB that account for the increased permeability to water with aging, recorded here.

Several studies report an increased expression of AQP4 to be associated with the aging process, with evidence of further enhancement in AD cases.^30^, ^31^, ^32^ This occurs together with a reduction of the AQP4 polarization at the perivascular location at the BBB that has been shown previously to correlate with the extent of cognitive decline in AD patients.^30^, ^31^ In the present study, the mRNA expression results lack spatial specificity and functional information. A comparison of the rates of vascular water exchange between a mouse model of AD and the aged mice, along with measurements of the location and function of the AQP4 water channels, would help to characterize the temporal cascade of events that occur at the BBB.

There were no measurable differences in cortical CBF detected with aging in the present study. A relatively subtle (13%) reduction of cerebral perfusion has been previously reported in aged mice (23‐month‐old compared with 6‐month‐old mice).^33^ Further longitudinal studies should be performed to investigate changes in the water permeability with age in relation to CBF reductions that have been previously reported in the aging brain. Also, this study reports no differences in the arterial transit time in the cortical brain region of the aged mice, which would suggest that the macrovascular properties are maintained as the brain ages. A potential limitation is that the arterial transit time model assumes that several parameters remain constant with age, such as arterial T_1_, which may change with aging. The arterial transit time was only measured in the cortical brain region, however it replicates the results from a previous study measuring the arterial transit times in different regions of the aging mouse brain.^34^

The multi‐TE ASL technique has potential as a clinical tool to measure BBB water permeability with aging in the human brain. In the present study the technique has been optimized for the cortical region of the mouse brain because of its relatively large size and proximity to the surface coil. A higher resolution readout would be necessary to target smaller brain regions, such as the hippocampus, which are associated with AD. To our knowledge, there have not been any techniques that have measured BBB permeability to water in the aging brain. Previous studies have measured an increased BBB permeability in the hippocampus and the caudate nucleus in the aging brain using contrast agent with DCE‐MRI techniques.^6^ Future studies could apply the multi‐TE ASL technique to the aging human brain to assess potential changes in BBB water exchange across different brain regions.

In summary, we have demonstrated evidence for compromised BBB integrity in the aging brain via noninvasive detection of increased BBB permeability to water. This finding indicates that an increase in BBB permeability to water occurs with age, which provides encouragement that ASL‐based BBB water permeability assessment techniques can noninvasively capture age‐related changes to the BBB that may play a key mechanistic role in the pathogenesis of neurodegenerative conditions such as AD.

## Supporting information

**FIGURE S1** ASL signal decay across the range of echo times (TE) at inflow times of 800 ms and 1500 ms, for individual adult mice (*n* = 9) with the mean value and the associated error (± standard deviation) indicated on each plot
**FIGURE S2** ASL signal decay across the range of echo times (TE) at inflow times of 800 ms and 1500 ms, for individual aged mice (*n* = 8) with the mean value and the associated error (± standard deviation) indicated on each plot
**FIGURE S3** A, Cortical arterial transit time measurements. B, Cortical cerebral blood flow (CBF) measurement in adult and aged mice. Each plot indicates the individual animal measurements along with mean value and the associated error (± standard deviation)

## APPENDIX 1

**TABLE A1:** Parameters that are measured from the data

	Measured variables	Units
Water exchange time	$T_{\mathrm{ex}}^{w}$	ms
Tissue transit time	*δ*	ms
Arterial transit time	*δ_α_*	ms
Cerebral blood flow	CBF	mL/100 g/min
Intravascular ASL signal	Δ*M*_IV_	–
Extravascular ASL signal	Δ*M*_EV_	–
Intravascular transverse relaxation time	*T*2_IV_	ms
Extravascular transverse relaxation time	*T*2_EV_	ms
Equilibrium magnetisation	M_0_	–

**TABLE A2:** Values of the assumed variables used in the models

	Assumed variables	Assumed value	Units
Blood‐brain partition coefficient	λ	0.9	–
Longitudinal relaxation rate (blood)	*R*1*_a_*	1/2.4	s^−1^
Longitudinal relaxation rate (tissue)	*R*1_app_	1/1.7	s^−1^
Temporal length of tagged bolus	τ	1.7	s
