# Supplementary material for: Increased blood–brain barrier permeability to water in the aging brain detected using noninvasive multi‐TE ASL MRI
Source: Magn Reson Med. 2020 Sep 10;85(1):326–33. doi: 10.1002/mrm.28496 (PMC8432141; doi:10.1002/mrm.28496)
Supplement: Supplementary file 1 — FIGURE S1 ASL signal decay across the range of echo times (TE) at inflow times of 800 ms and 1500 ms, for individual adult mice (n = 9) with the mean value and the associated error (± standard deviation) indicated on each plot FIGURE S2 ASL signal decay across the range of echo times (TE) at inflow times of 800 ms and 1500 ms, for individual aged mice (n = 8) with the mean value and the associated error (± standard deviation) indicated on each plot FIGURE S3 A, Cortical arterial transit time measurements. B, Cortical cerebral blood flow (CBF) measurement in adult and aged mice. Each plot indicates the individual animal measurements along with mean value and the associated error (± standard deviation) [file MRM-85-326-s001.docx]

## Supporting Information

##


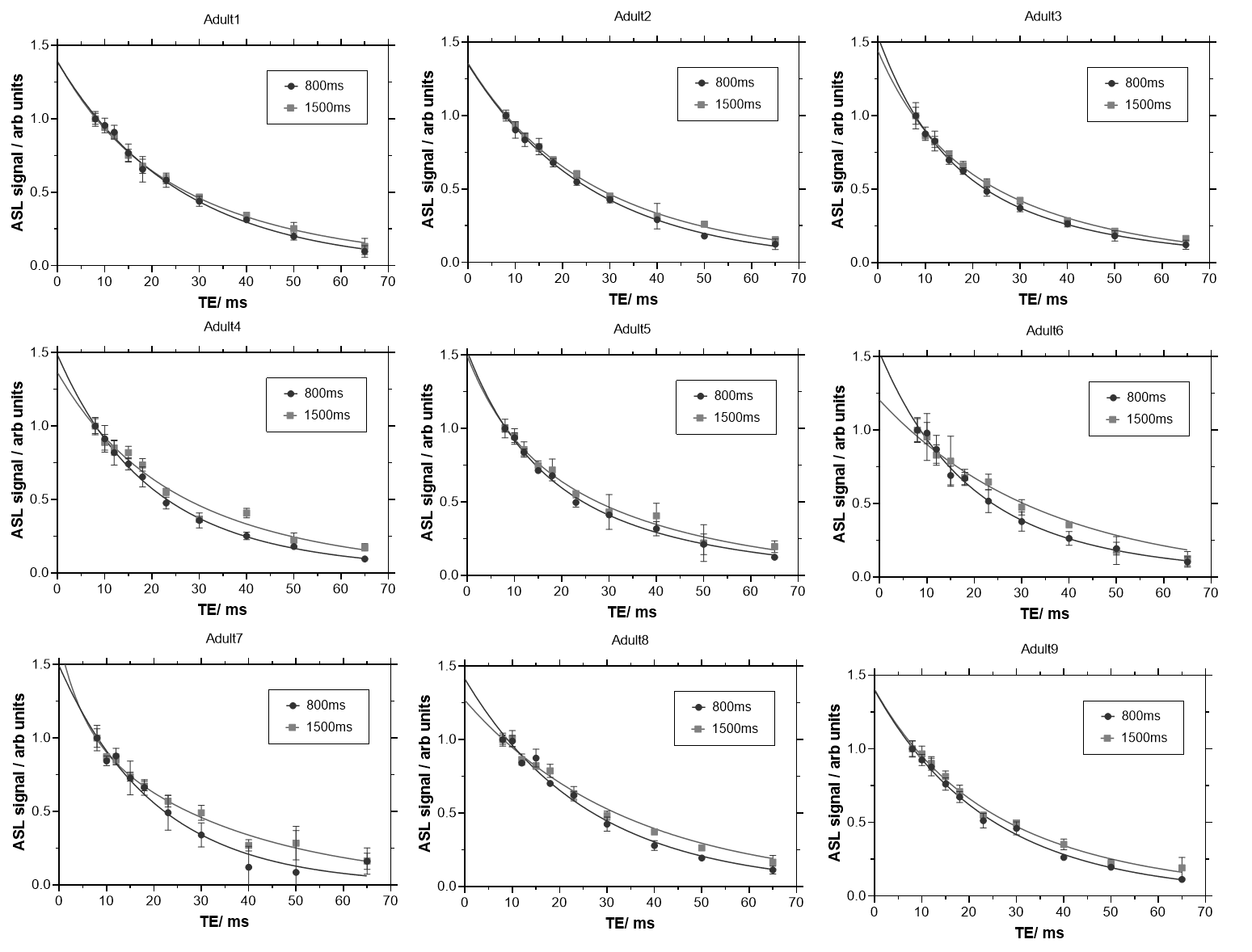


**Supporting Information Figure S1:** ASL signal decay across the range of echo times (TE) at inflow times of 800ms and 1500ms, for individual adult mice (n = 9) with the mean value and the associated error (± standard deviation) indicated on each plot.


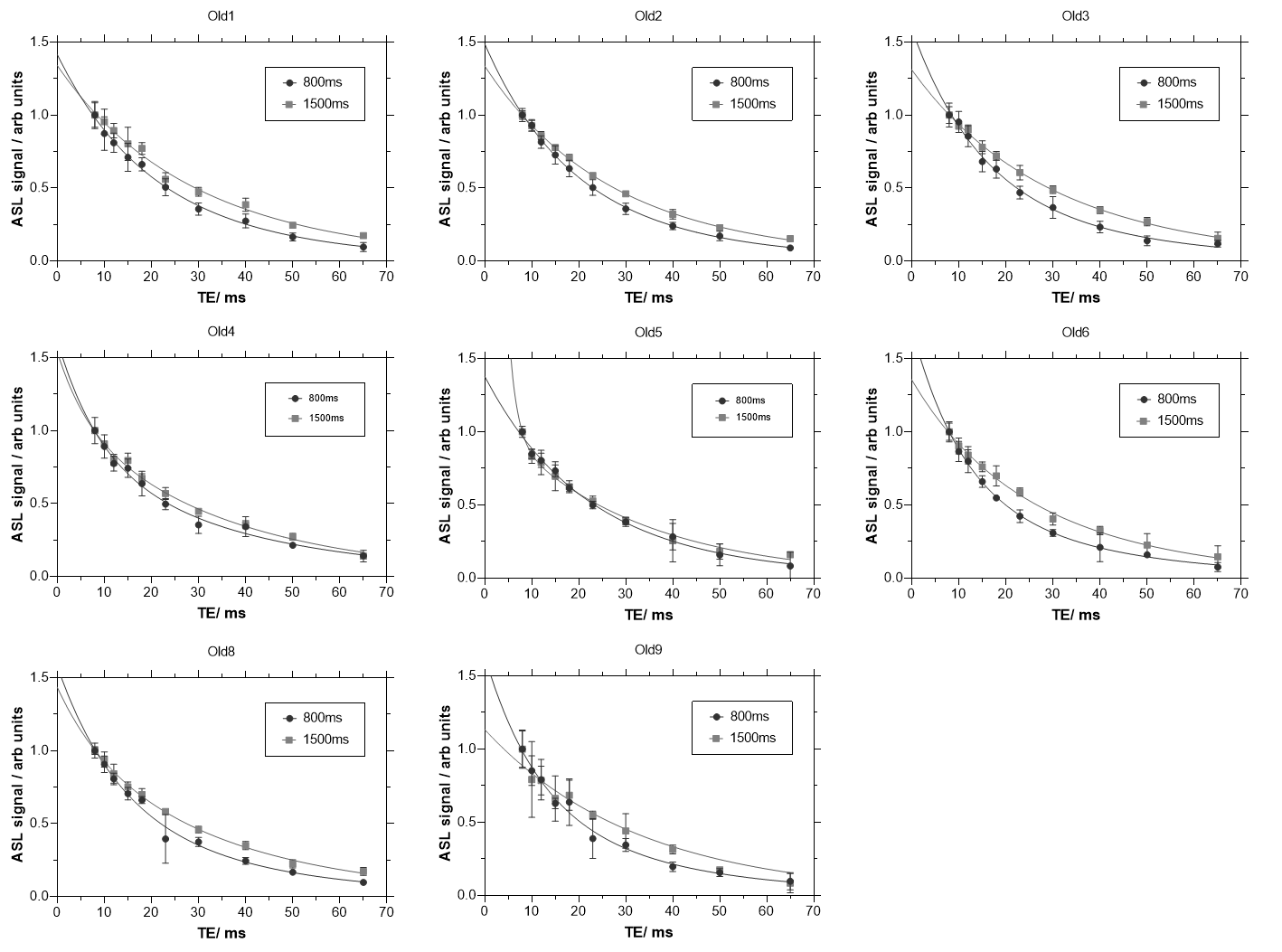


**Supporting Information Figure S2:** ASL signal decay across the range of echo times (TE) at inflow times of 800ms and 1500ms, for individual aged mice (n = 8) with the mean value and the associated error (± standard deviation) indicated on each plot.


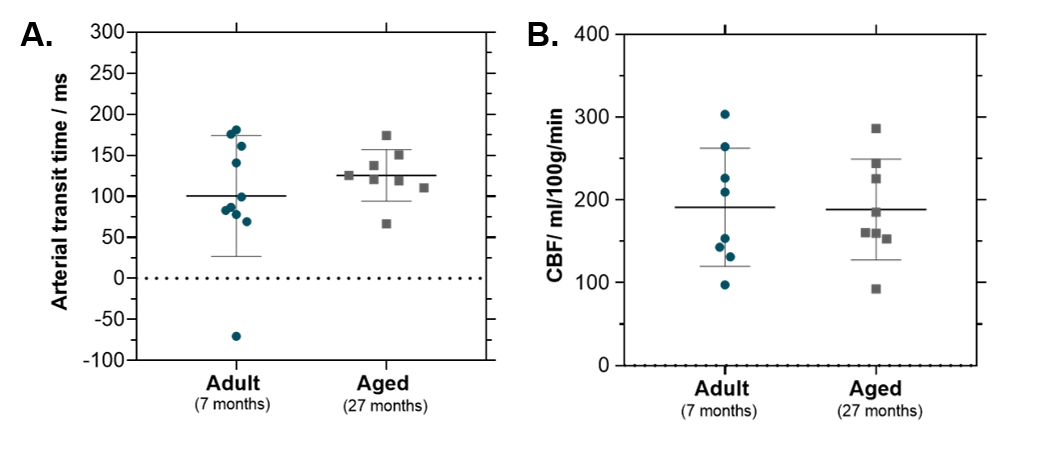


**Supporting Information Figure S3: A.** Cortical arterial transit time measurements **B.** Cortical cerebral blood flow (CBF) measurement in adult and aged mice. Each plot indicates the individual animal measurements along with mean value and the associated error (± standard deviation).
